# Association Between the Frequent Use of Perineal Talcum Powder Products and Ovarian Cancer: a Systematic Review and Meta-analysis

**DOI:** 10.1007/s11606-022-07414-7

**Published:** 2022-02-02

**Authors:** Sean A Woolen, Ann A. Lazar, Rebecca Smith-Bindman

**Affiliations:** 1 Department of Radiology and Biomedical Imaging, University of California San Francisco, San Francisco, CA USA; 2 Department of Preventive and Restorative Dental Sciences, University of California San Francisco, San Francisco, USA; 3 Department of Epidemiology and Biostatistics, University of California San Francisco, San Francisco, USA; 4 Department of Obstetrics, Gynecology and Reproductive Sciences, University of California San Francisco, San Francisco, USA; 5 Philip R Lee Institute for Health Policy Studies, University of California San Francisco, San Francisco, USA

**Keywords:** talcum powder, ovarian neoplasm, women’s health, meta-analysis

## Abstract

**Background:**

Risk of ovarian cancer in women with frequent perineal talcum powder product is not well understood. Prior systematic reviews focused on ever use. The purpose of this study is to estimate the association between frequent (at least 2 times per week) perineal talcum powder use and ovarian cancer.

**Methods:**

A systematic review and meta-analysis was conducted according to meta-analysis of observational studies in epidemiology guidelines. Study protocol was prospectively registered at PROSPERO (registration number CRD42020172720). Searches were performed in PubMed, Embase, Web of Science, and Cochrane Central Register of Controlled Trials databases from their inception to August 2, 2021. Case-control and cohort studies were included if they reported frequent perineal talcum powder use and an adjusted odds ratio or hazard ratio for ovarian cancer. Review for inclusion, data extraction, and quality assessment (using the Newcastle-Ottawa Scale [NOS]) were performed independently by two reviewers. Pooled adjusted odds ratios with 95% confidence intervals were generated from the random effects model. Heterogeneity was quantified with *I*^2^ statistic. Funnel plot and Eger’s test were performed to assess publication bias. Subgroup and sensitivity analyses were performed for testing the robustness of the overall findings.

**Results:**

Initial database searches returned 761 unique citations and after review, eleven studies describing 66,876 patients, and 6542 cancers were included (Cohen’s *κ* = 0.88). Publication quality was high (median NOS = 8, range: 4 to 9). Frequent talcum powder use was associated with an elevated risk of ovarian cancer (adjusted pooled summary odds ratio 1.47 (95% CI 1.31, 1.65, *P*<0.0001). There was no evidence of bias and low heterogeneity (*I*^2^= 24%, *P*=0.22). There was no meaningful difference limiting analysis to publications with a NOS quality score of 8 or 9 or limiting studies based on study design.

**Conclusions:**

This review suggests an increased risk of ovarian cancer associated with frequent perineal powder exposure of 31–65%.

**Supplementary Information:**

The online version contains supplementary material available at 10.1007/s11606-022-07414-7.

## INTRODUCTION

Talc, the primary ingredient in baby powder, is a naturally occurring mineral known for its softness and absorbency and has been added to a broad range of personal care products since the early part of the twentieth century. To date, there have been seven systematic reviews and pooled data studies^1–7^ of the relationship between talcum powder products and ovarian cancer. All studies including retrospective case-control studies have found a positive association.^2–7^ The most recent review by O’Brien^1^ limited analysis to the four prospective cohort studies and in its main conclusion stated there was no statistically significant association between genital talc use and ovarian cancer.

The differences in conclusions between the metanalyses are at least partially due to the inconsistent talcum-based powder exposure questions regarding the frequency and type of exposure. To harmonize the exposure measurements across the greatest number of studies, prior meta-analyses primarily focused on quantifying the association between ever versus never use of talcum powder products and ovarian cancer. However, ever use is a non-specific exposure that could dilute or obscure a meaningful association as ever use would combine women with low and high exposures to talcum powder.

Knowledge of ovarian cancer risks are important for women’s health and important to guide regulatory oversight. The purpose of this analysis is to estimate the risk of ovarian cancer associated with the frequent use of talcum powder products. We hypothesize that assessment of ovarian cancer risk among frequent users of talcum powder products would provide a more meaningful assessment of its carcinogenicity.

## METHODS

We conducted a systematic review of the literature and meta-analysis to assess the association between the frequent perineal exposure to talc and ovarian cancer following the Meta-analyses of Observational studies (MOOSE) reporting guidelines. A study protocol was developed in advance and registered with the International Prospective Register of Systematic Reviews (PROSPERO) on April 28, 2020 (registration number: CRD42020172720).

### Search Strategy and Information Sources

Comprehensive searches were performed by an expert health science informationist from inception of the relevant databases to August 2, 2021. Searches were completed in the following: PubMed, Embase, Web of Science, and Cochrane Central Register of Controlled Trials. Each search consisted of talcum powder and ovarian cancer concept blocks, which can be viewed in the supplemental material. No date, language, or other restrictions were incorporated into the searches. Duplicate citations were removed in Endnote X9.3.1 (Clarivate Analytics). The references of all publications were searched to identify additional publications.

### Eligibility Criteria and Study Selection

Selection of studies included observational cohort and case-control study designs. Studies were included if they reported primary data on frequent, defined as multiple (2 or more) times per week perineal exposure to talc, and reported an adjusted odds ratio or hazard ratio and 95% confidence interval (CI) for ovarian malignancy. Other uses of talcum powder, including applications to the body, were excluded to isolate exposure mechanism to perineal application. Conference abstracts, retracted manuscripts, narrative reviews, editorials, case reports, and manuscripts not reporting the location or frequency of talcum powder application were excluded.

**Figure 1.**
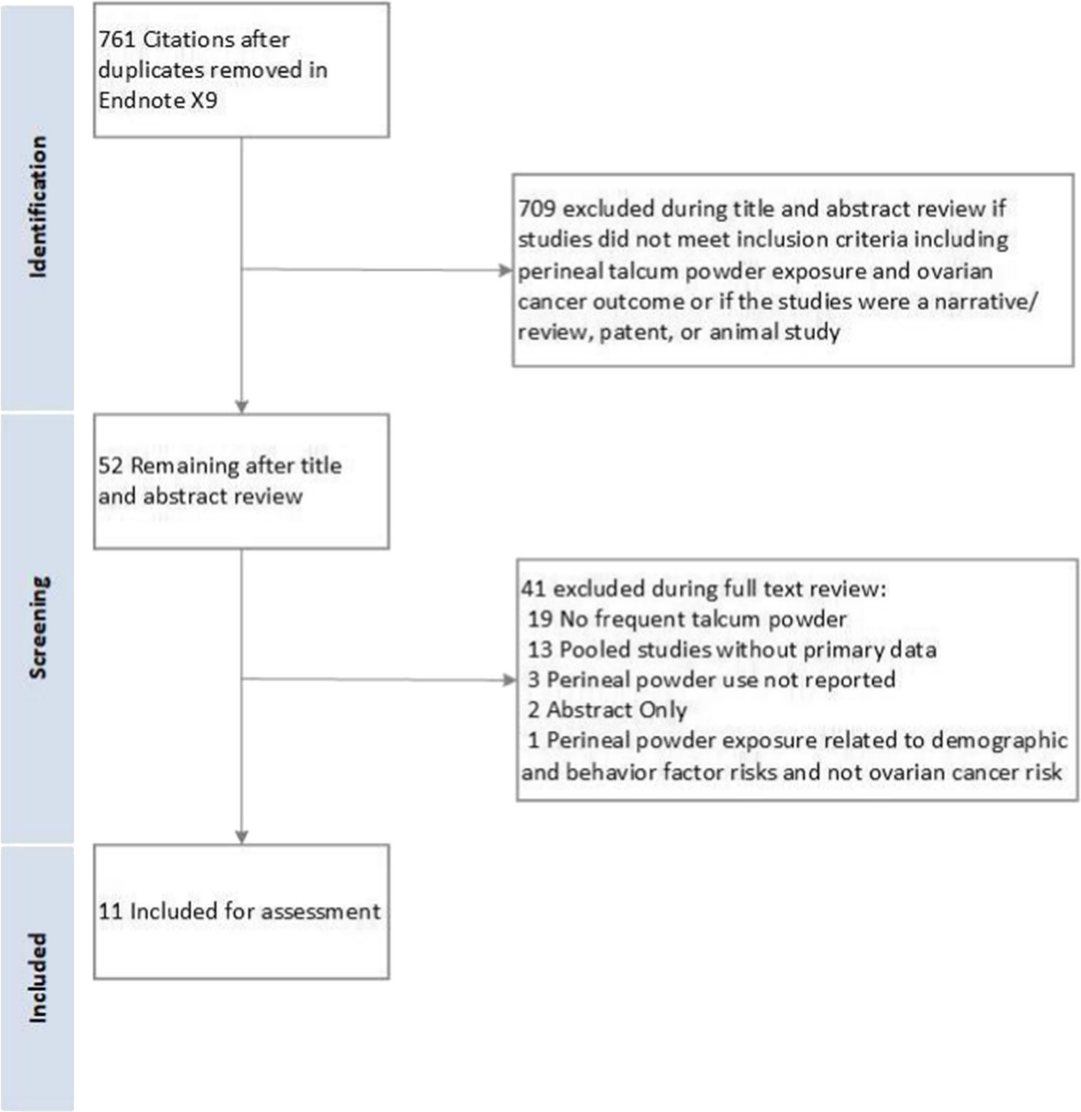
Flow diagram of study selection.

Studies were screened for inclusion using prespecified selection criteria by a single author (SW). Selection criteria included publication of primary data, reporting on multiple times per week (≥ 2 times per week) perineal exposure to talcum powder including direct application of talcum powder to the perineum and rectum, application to underwear or sanitary napkins, or on birth control devices like diaphragms and risk for ovarian malignancy. Studies were also selected for baseline quality requiring a multivariable risk adjustment, study size (*n*>10 cancers), and defined research methods to allow assessment of inclusion and exclusion criteria (flow diagram Fig. 1). The search resulted in 761 citations, which were screened at the title and abstract level by a single author (SW). The full manuscripts of relevant citations (*n*=52) were independently reviewed by two authors (SW and RSB). Agreement for inclusion was calculated using the Cohen *k* with the following scale: 0.01 to 0.20 indicates slight; 0.21 to 0.40, fair; 0.41 to 0.60, moderate; 0.61 to 0.8, substantial; and 0.81 to 0.99, almost perfect. Disagreements were resolved by discussions.

The four prospective cohort studies as reported in O’Brien^1^ did not meet the pre-specified definition for frequent exposure. However, the questionnaires for two of the included cohorts had asked women about more frequent talcum powder use. As is standard in systematic reviews to include relevant but unpublished results, we contacted O’Brien and requested primary data from the Nurses’ Health Study 1 (NHS1) and the Sisters Study (SIS) for the highest frequency talc exposure group. The data from NHS 1 were provided and described in the Supplemental Table 1 and are included in the systematic review. The data from the SIS study were not provided to us due to the small sample size of exposed individuals in the highest exposure category (*n*=2 women).

### Data Extraction

Data extraction was performed by two authors (SW and RSB). Extracted information included age range, enrollment period, definition of frequency of talc use, size of control group or cohort size, number of ovarian cancers, and adjusted hazard ratio and odds ratio with 95% CI. Data were included from the highest reported talc use category to obtain as close to daily use as possible and the referent group were women who reported no talc exposure. When duplicate reports of the same subjects were published, the publication reporting the highest talc use was selected. The senior author was contacted to obtain data for studies that reported frequent perineal talcum powder exposure but did not publish the results on this exposure. Disagreements in data extraction were resolved by consensus.

### Assessment of Risk of Bias and Study Quality

Eleven articles met selection criteria. The articles were independently reviewed by two authors (SW and RSB) for quality using the Newcastle Ottawa Scale (NOS) where a score of 0–3 reflects a very high risk of bias, 4–6 reflects a high risk of bias, and 7–9 reflects a high-quality study with low risk of bias. The scale assesses the criteria used to select study groups, the comparability of study groups, and ascertainment exposure or outcome of interest. Results of the validity assessment were discussed until agreement was reached (Table 1).

**Table 1.** Quality Assessment of Included Studies Using the Newcastle-Ottawa Scale

Case-control design*		**Selection**	**Comparability**	**Exposure**	**Total score (out of 9)**
	Booth et al., 1989^10^	**	**	-	4
	Chang et al., 199 [11]	****	**	**	8
	Cook et al., 1997^12^	****	*	**	7
	Cramer et al., 2016^13^	****	**	**	8
	Harlow et al., 1992^14^	****	**	**	8
	Mills et al., 2004^15^	****	**	**	8
	Rosenblatt et al., 2011^16^	****	**	**	8
	Schildkraut et al., 2016^17^	****	**	**	8
	Whittenmore et al., 1988^18^	***	**	**	7
	Wu et al., 2009^19^	****	**	**	8
**Cohort design**				**Outcome**	
	O’Brien (National Heath Study 1), 2020^1^	****	**	***	9

Each asterisk denotes 1 point. The empty cells indicate the study received no points in the category.

All the case-control studies lost a point in the exposure category because they did not report if the interviewers were blinded to cancer status when the interviews were conducted.

### Data Synthesis

Individual study results were combined using summary estimates generated from the random effects model^8^ and displayed with forest plots. Adjusted odds ratios and the adjusted hazard ratio were combined given the infrequency of ovarian cancer.^9^ As a sensitivity analysis, we removed the one cohort study from the analysis and a study that combined non-perineal talcum powder users with perineal talcum powder users.

Heterogeneity was evaluated by inspecting funnel plots, and by calculating the *I*^2^ values. Publication bias was evaluated by visual inspection of the funnel plot and statistically with Egger’s test. The NOS score was calculated for each study and median, maximum, and minimum scores were calculated across all included studies. As sensitivity analyses, we repeated the meta-analysis first excluding the study with the NOS score of 4, and then repeated the analysis limited to studies with an NOS score of 8 or 9. SAS v. 9.4 was used, and two-sided*P*-values less than 0.05 were considered statistically significant.

## RESULTS

Initial database searches returned 761 unique citations (Fig. 1). After title and abstract review, 52 potential citations remained. After full-text review, 11 publications met the inclusion criteria including a total of 6542 ovarian cancer cases and 66,876 women (Table 2).^1,10–19^ The interrater agreement in determining the final study cohort from the 52 full-text reviews was excellent (Cohen’s *k*=0.88). The included studies include 10 retrospective case-control studies and a single cohort study. The median NOS of included case-control studies was 8 (range: 4 to 8). The NOS of the included cohort study was 9.

**Table 2.** Publications Included in the Systematic Review. The Most Frequent Perineal Talcum Powder Use Reported for Each Study Was Abstracted

	**First author**	**Study type**	**Year**	**Age range**	**Enrollment period**	**Specification of talc exposure**^(1)^	**Ovarian cancer cases**		**Controls or cohort**	
							No. Exposed	No. Total	No. Exposed	No. Total
1	Booth M^10^	Case-control	1989	20–64	1978–1983	Daily	71	217	139	434
2	Chang S^11^	Case-control	1997	35–79	1989–1992	> 25× per month	41	450	60	564
3	Cook LS^12^^(1)^	Case-control	1997	20–79	1986–1988	> 10,000 lifetime	28	313	17	422
4	Cramer DW^13^	Case-control	2016	18–80	1992–1997 1995–2002 2003–2008	> 30× per month	267	2041	205	2100
5	Harlow BL^14^^(2)^	Case-control	1992	18–76	1984–1987	>10,000 lifetime	58	235	41	239
6	Mills PK^15^^(1)(3)^	Case-control	2004	41–70	2000–2001	4–7× per week	41	249	122	1100
7	Rosenblatt KA^16^^(1)^	Case-control	2011	35–74	2002–2005	> 10,000 lifetime	18	812	37	1313
8	Schildkraut JM^17^^(1)(3)^	Case-control	2016	20–79	2010–2015	Daily	158	582	134	744
9	Whittemore AS^18^	Case-control	1988	18–74	1983–1985	> 20× per month	44	188	101	539
10	Wu AH^19^^(4)^	Case-control	2009	18–74	1998–2002	> 30× per month and > 20 years	67	605	45	688
11	O’Brien (NHS 1)^1^^(5)^	Cohort	2020	35–62	1982–2016	Daily	157	850	355	52191

*NHS* Nurses’ Health Study

For each study that specified the number of women who did not respond to talc questions, these women were subtracted from the total number of cases and controls.

^(1)^Cook, Mills, Rosenblatt, and Schildkraut did not differentiate between talc and cornstarch powders. Cornstarch is estimated to reflect 1–2% of powder

^(2)^Harlow reports an adjusted odds ratio for daily talcum powder exposure, and for > 10,000 lifetime uses. The point estimates are the same for each and the 95% CI almost identical. We include data for > 10,000 lifetime uses as this number is explicitly defined as perineal exposure

^(3)^Shildkraut was the only study that included women recruited after two class action lawsuits were filed in 2014 concerning possible carcinogenic effects of body powder influencing recall of use. The study adjusted for individuals answering questions after 2014 to account for increased recall bias

^(4)^Wu combined non-perineal with perineal exposures. Wu reported an adjusted odds ratio for women who used talcum powder > 30× per month and > 20 years

^(5)^O’Brien did not publish on daily exposure for the National Health Study participants. However, these data were available and O’Brien provided these data for inclusion. The entirety of the data we were provided are shared in the supplementary table. We include data on women with intact fallopian tubes, to harmonize with other publications

**Figure 2.**
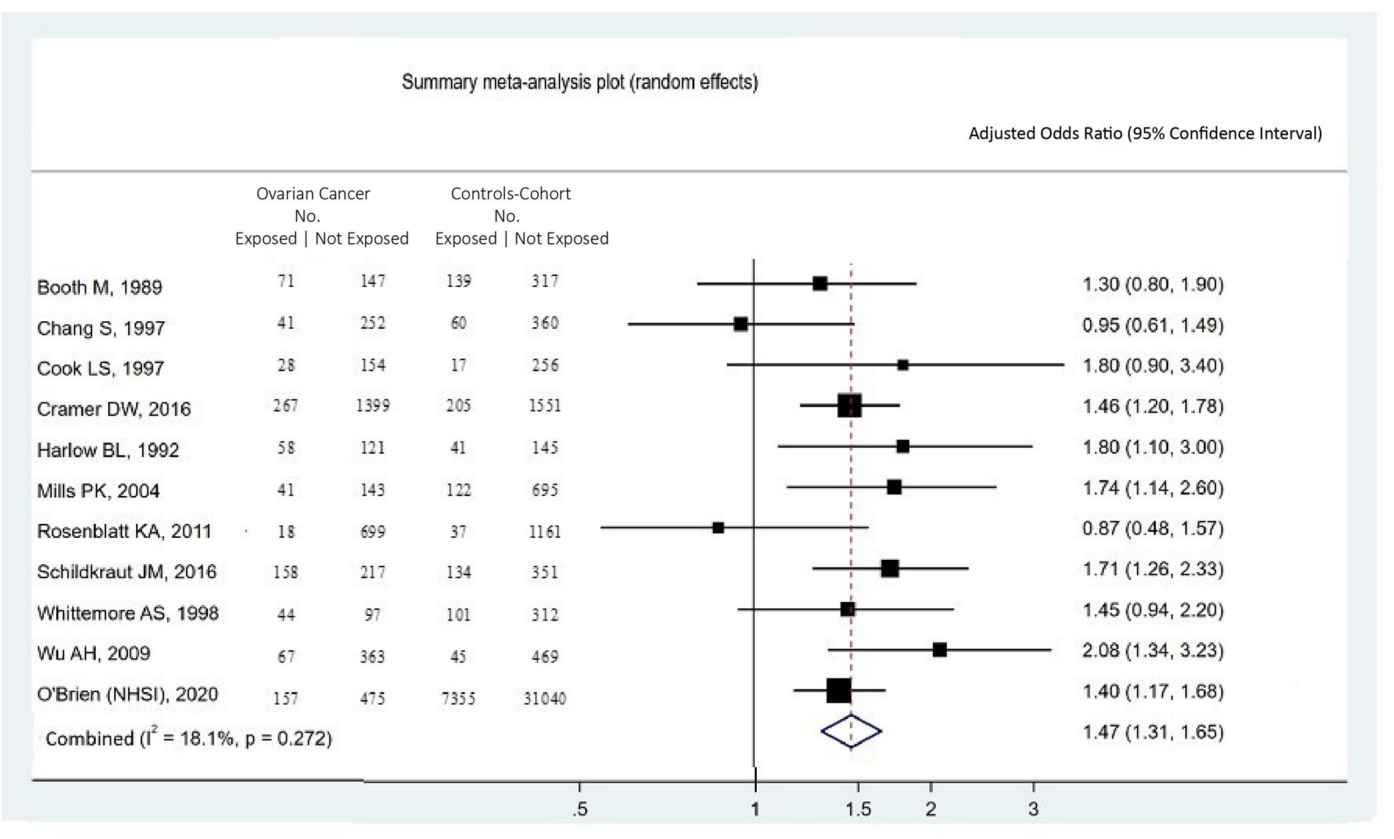
Forest plot showing the summary meta-analytic estimate for the association between frequent use of perineal talcum powder products and the risk of ovarian cancer. The number (No.) of women included as cases and controls (or cohort) who were exposed and not exposed are provided (excluding from the table women who had exposure to talcum powder at less than the highest exposure). The study-specific odds ratios and 95% confidence interval are on the right side of the plot.

**Figure 3.**
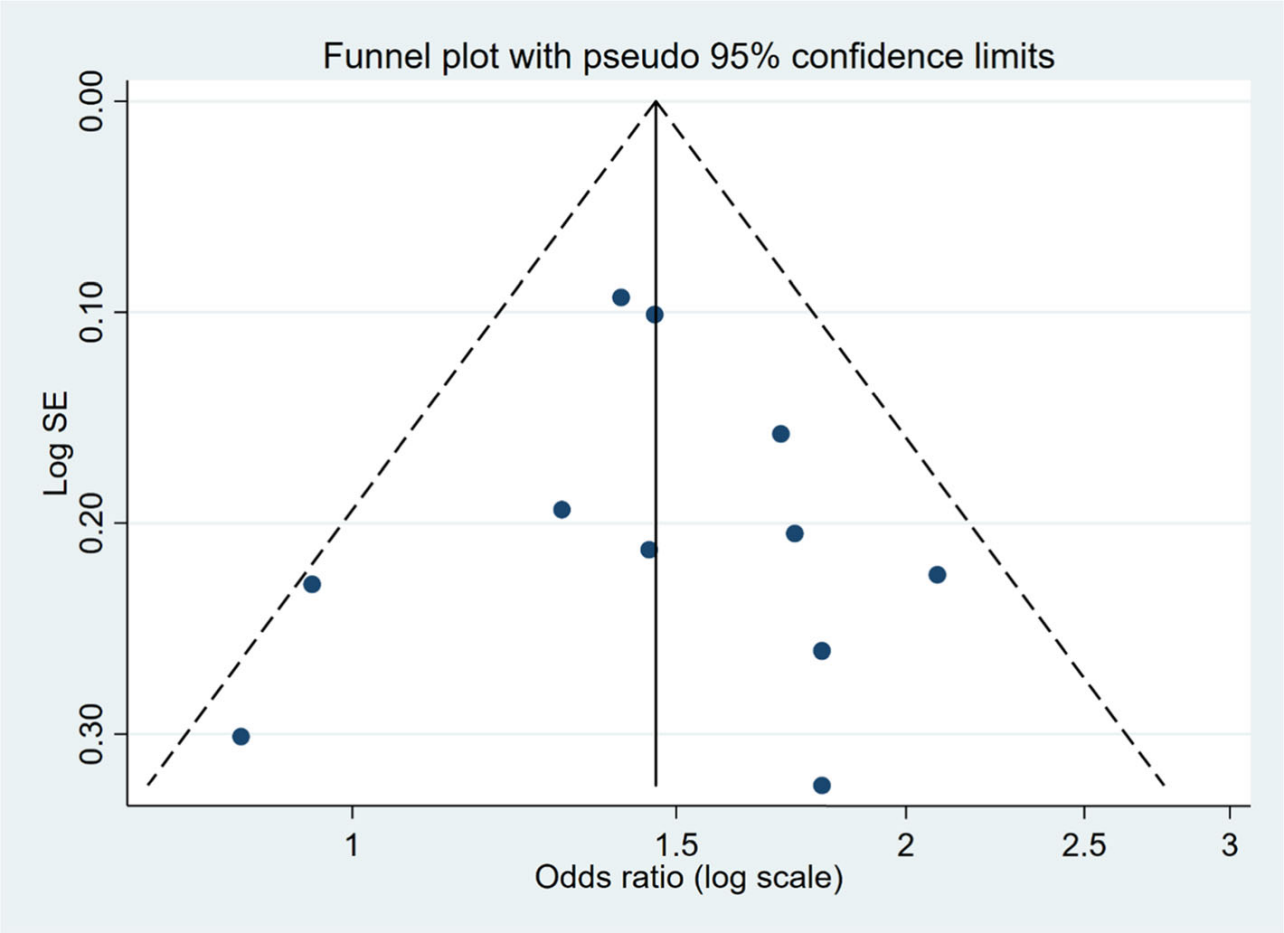
Funnel plot for the risk of publication bias.

The age range of included women was 18–79 years. Studies were published between 1988 and 2016. Among included studies, the range of frequent talcum powder use was defined as 4–7× per week, and 45% (5 of 11) reported daily exposure. The studies were homogeneous, and 24.4% (*P*=0.22) of the variation across studies were due to heterogeneity. The summary pooled odds ratio assessing the association between frequent use of perineal talcum powder products and ovarian cancer was 1.47 (*P*<0.0001, 95% CI 1.31, 1.65) (Fig 2) and there was no significant publication bias (Egger's test, P=0.94) (Fig 3). When limited to the case-control studies, the summary pooled odds ratio was 1.49 (*P*<0.0001, 95% CI 1.29, 1.72) (Supplementary Figures 1 and 2), whereas the odds ratio for the cohort study was 1.40 (95% CI 1.17, 1.68). When excluding Wu et al.^19^ which combined perineal administration of talcum powder with other methods, the summary pooled odds ratio was 1.44 (95% CI 1.29, 1.60) (Supplementary Figure 3) and the studies remained homogenous (*I*^2^= 6.5%, *P*=0.382) without publication bias (Egger’s test, *P*=0.77) (Supplementary Figure 4).

Given the quality of a meta-analysis is dependent on the quality of the studies it includes, we conducted a sensitivity analysis excluding the study with a NOS grade of 4 (Booth^10^). There was no meaningful change after excluding Booth, summary pooled odds ratio 1.48 (*P*<0.0001, 95% CI 1.31 to 1.69) (Supplementary Figure 5) and the studies remained homogenous (*I*^2^= 24.4%, *P*=0.22) without publication bias (Egger’s test, *P*=0.88) (Supplementary Figure 6). The second sensitivity analysis was restricted to studies with a NOS of 8 or 9, removing Booth et al.^10^ (NOS=4), Cook et al.^12^ (NOS=7), and Whittemore et al.^18^ (NOS=7), also without a statistically significant change in the summary pooled odds ratio of 1.48 (*P*<0.0001, 95% CI 1.27 to 1.72) (Supplementary Figure 7). The studies remained homogenous (*I*^2^=39.3%, *P*=0.12) and did not have publication bias (Egger’s test, *P*=0.97) (Supplementary Figure 8).

## DISCUSSION

We found frequent use of perineal talcum powder is associated with an increased risk of ovarian cancer, with a pooled adjusted odds ratio of 1.47 (95% CI 1.31, 1.65). The 11 contributing studies included in the review, including the longest published follow-up available from the Nurses’ Health Study cohort, were homogenous, and the summary estimate was robust in the sensitivity analysis. This meta-analysis makes a significant contribution to the available evidence as it is the first study that summarizes the published cohort and case-control literature that focuses on frequent (multiple times per week) rather than ever use of talc.

The precise mechanism whereby talcum powder causes ovarian cancer is not fully understood. It is widely speculated that trans-genital migration of talc powder through the fallopian tubes to the ovaries and peritoneum results in inflammation and a cascade of changes that result in carcinogenesis.^20,21^ Talc fibers are found within normal ovaries and within ovarian cancer with “cosmetic” talc use^22,23^ supporting this theory. A case series by Steffen et al. reported 10 cases of patients with “cosmetic” talc exposure and serous ovarian cancer evaluated the surgical specimens as well as cosmetic talcum powder manufactured in the 1950s. The transmission electron microscope tissue analysis and phase contrast microscopy found talc in every serous ovarian cancer and anthophyllite asbestos in 80% (8/10) of the serous ovarian cancers, which matched the talcum-based powder from the 1950s. Asbestiform talc is considered, along with asbestos, as a class 1 carcinogen for ovarian cancer, by the International Agency for Research on Cancer.^24^ Thus, the strongest and most widely believed theory is that asbestiform talc fibers and asbestos fibers are the etiological agents leading to the elevated cancer risk associated with their use.

The pooled odds ratio of 1.47 is higher than that of six additional systematic reviews which included case-control studies, all of which also report a statistically significant association between talc and ovarian cancer (range of adjusted odds ratios: 1.24–1.35).^2–7^ O’Brien et al.^1^ concluded that perineal talcum powder exposure in cohort studies was not significantly associated with ovarian cancer. However, when O’Brien limited to women with patent fallopian tubes the hazard was 1.13 (95% CI: 1.01 to 1.26) for ever versus never use and there was a dose response with increasing risk with increasing frequency of talcum powder use with a hazard ratio of 1.40 (95% CI: 1.17 to 1.68) for daily users. The magnitude of the higher association in our study compared to prior case-control and cohort meta-analyses was likely due to our focus on frequent rather than any talcum powder users, inclusion of quality studies, and consistent definition of the exposure.

Our meta-analysis included both case-control and cohort observational studies allowing use to include more cancer cases than cohort alone. Although there is the general belief that cohort studies are better than case-control studies, both can provide accurate and meaningful information about statistical associations. Since most publications on talc used a case-control design with detailed quantification of the type and frequency of talc exposure missing from most of the cohort studies, it is important to include the data from the case-control studies in any summary estimate of the association with ovarian cancer. While cohort studies can provide a superior design for some research questions, the method provides less useful results when quantification of the exposure is too crude to provide a meaningful estimation of exposure. Cohort studies for talcum powder did not define the type of powder exposure, consistently define frequency and duration, capture changes in exposure over time, and relied on recall by participants for exposure which may involve misclassification as would be expected in case control studies.^25–27^ Although it is well known that case-control study design is susceptible to recall bias and misclassification, the bias was likely limited to participant recollection of their exposure rather than influenced by the media, given there was no publicity on the topic at the time of most publications: the first legal case was reported in the press in 2014 long after most studies completed recruitment. The one study where patient recruitment occurred after 2014 patients was Schildkraut et al.,^17^ where recruitment occurred between 2010 and 1015, and they adjusted for the additional bias.

### Strengths and Limitations

The primary strength of our study is our focus on frequent users of perineal talcum powder. Among women who report talcum powder use, the most common frequency is daily use,^13^ and this is the first systematic review to focus on multiple times per week users. The results were highly consistent and homogenous, and the included studies were of high quality. The work has limitations as well. We constructed our selection criteria prospectively to include studies with multiple times per week and as close to daily talcum powder exposure as possible. However, this meant that cohort and case-control studies that might have frequent-use patients were excluded if the questionnaire did not explicitly capture this information. The definition of talcum powder use varied by frequency and duration between the case-control and cohort studies. Additionally, studies by Cook et al.^12^, Mills et al.^15^, Rosenblatt et al.^16^, and Schildkraut et al.^17^ were unable to differentiate between use of perineal powders and the small subset using cornstarch (estimated at 1.5%). However, the differences in definition and small inclusion of cornstarch likely did not affect the results as there was no evidence for statistical heterogeneity in our study. The included studies were adjusted for multiple covariates. The possibility of additional confounders to the studies likely exists.

### Conclusions and Implications

In this analysis of pooled data from 10 case-control studies and a single cohort study, the frequent use of perineal talcum powder use is associated with increased risk of ovarian cancer. These results support women avoiding the frequent use of talcum powder in the perineal area.

## Supplementary Information

ESM 1 (DOCX 13883 kb)
