# Supplementary material for: Association Between the Frequent Use of Perineal Talcum Powder Products and Ovarian Cancer: a Systematic Review and Meta-analysis
Source: J Gen Intern Med. 2022 Feb 2;37(10):2526–32. doi: 10.1007/s11606-022-07414-7 (PMC9360263; doi:10.1007/s11606-022-07414-7)

**Supplementary Table 1.** Hazard Ratios and 95% Confidence Intervals for the Association between Frequency of Genital Powder Use and Risk of Ovarian Cancer in the Nurses’ Health Study

|  | **Person-time at risk**^a^ | **Non-cases^a^** | **Ovarian**  **cancer cases^a^** | **Crude Hazard Ratio** (95% CI) | **Adjusted Hazard Ratio**^b^ (95% CI) |
| --- | --- | --- | --- | --- | --- |
| **All women** |  |  |  |  |  |
| Non-users | 1,263,610 | 46,786 | 706 | 1.00 | 1.00 |
| Less frequent users | 566,785 | 20,979 | 302 | 0.96 (0.84, 1.09) | 0.96 (0.84, 1.10) |
| Daily users | 300,402 | 11,290 | 216 | 1.27 (1.09, 1.48) | 1.27 (1.09, 1.49) |
| **Women with Patent Fallopian Tubes** |  |  |  |  |  |
| Non-users | 838,445 | 31,040 | 475 | 1.00 | 1.00 |
| Less frequent users | 373,969 | 13,796 | 218 | 1.03 (0.88, 1.21) | 1.04 (0.88, 1.68) |
| Daily users | 196,578 | 7,355 | 157 | 1.40 (1.17, 1.67) | 1.40 (1.17, 1.68) |

^a^Among participants with complete covariate information. Includes all self-reported cases.

^b^Hazard ratios are adjusted for race/ethnicity (white, black, other), education <high school, some college, >college graduate), BMI (as a restricted cubic spline), parity (0, 1, 2, 3+ births), ever oral contraceptive use, tubal ligation (yes/no), hysterectomy status (yes/no), menopausal status (pre or post-menopausal), ever hormone therapy use. All covariates correspond to status at time of genital powder assessment.

^f^Patency defined as having a uterus (i.e. no hysterectomy) and not having had a tubal ligation

**Supplementary Figure 1**. Forest Plot Showing the Summary Meta-analytic Estimate for the Association Between Frequent Use of Perineal Talcum Powder Products and The Risk of Ovarian Cancer after removing single cohort study. The pooled odds ratios and 95% confidence interval are on the right side of the plot.

**
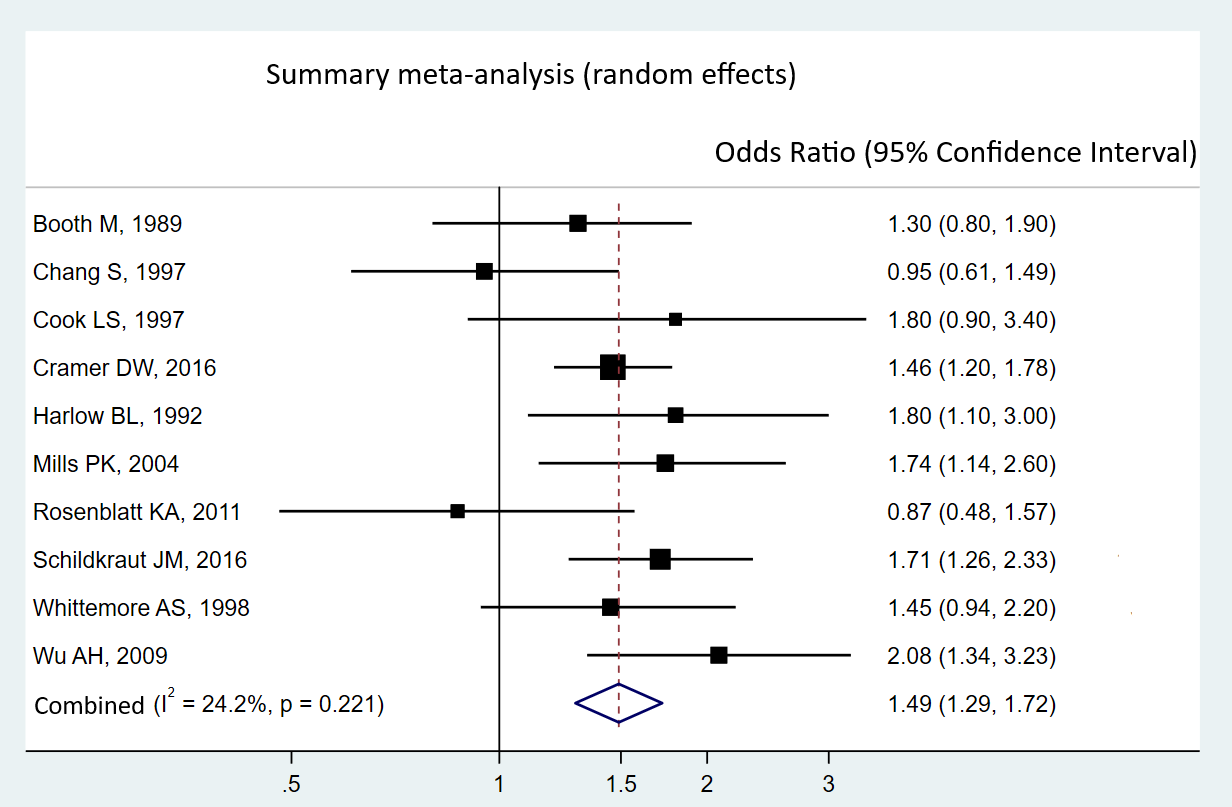
**

**Supplementary Figure 2.** Funnel plot for the risk of publication bias after removing single cohort study.

**
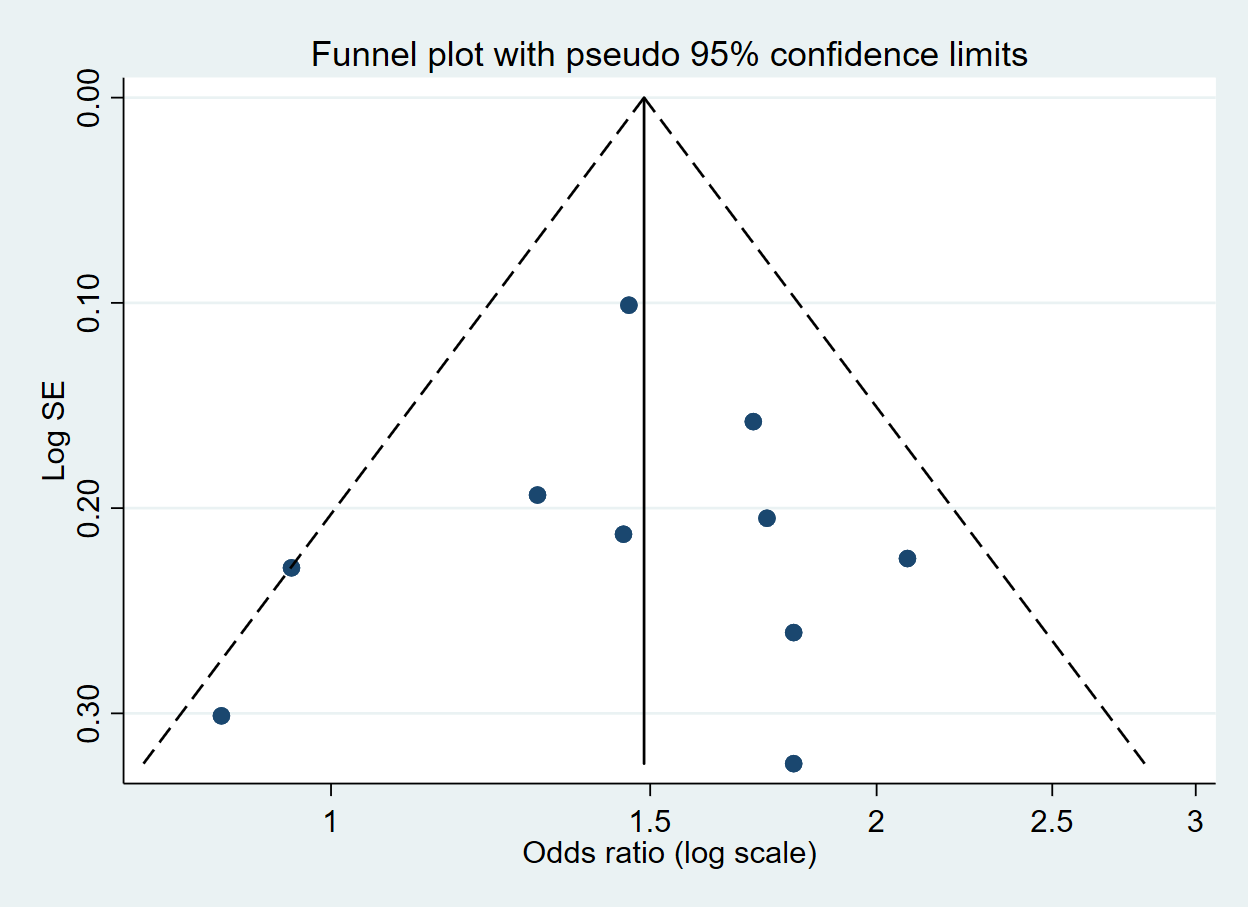
**

**Supplementary Figure 3**. Forest Plot Showing the Summary Meta-analytic Estimate for the Association Between Frequent Use of Perineal Talcum Powder Products and The Risk of Ovarian Cancer after removing Wu et al which combined perineal exposure with a small number of non-perineal users. The pooled odds ratios and 95% confidence interval are on the right side of the plot.

**
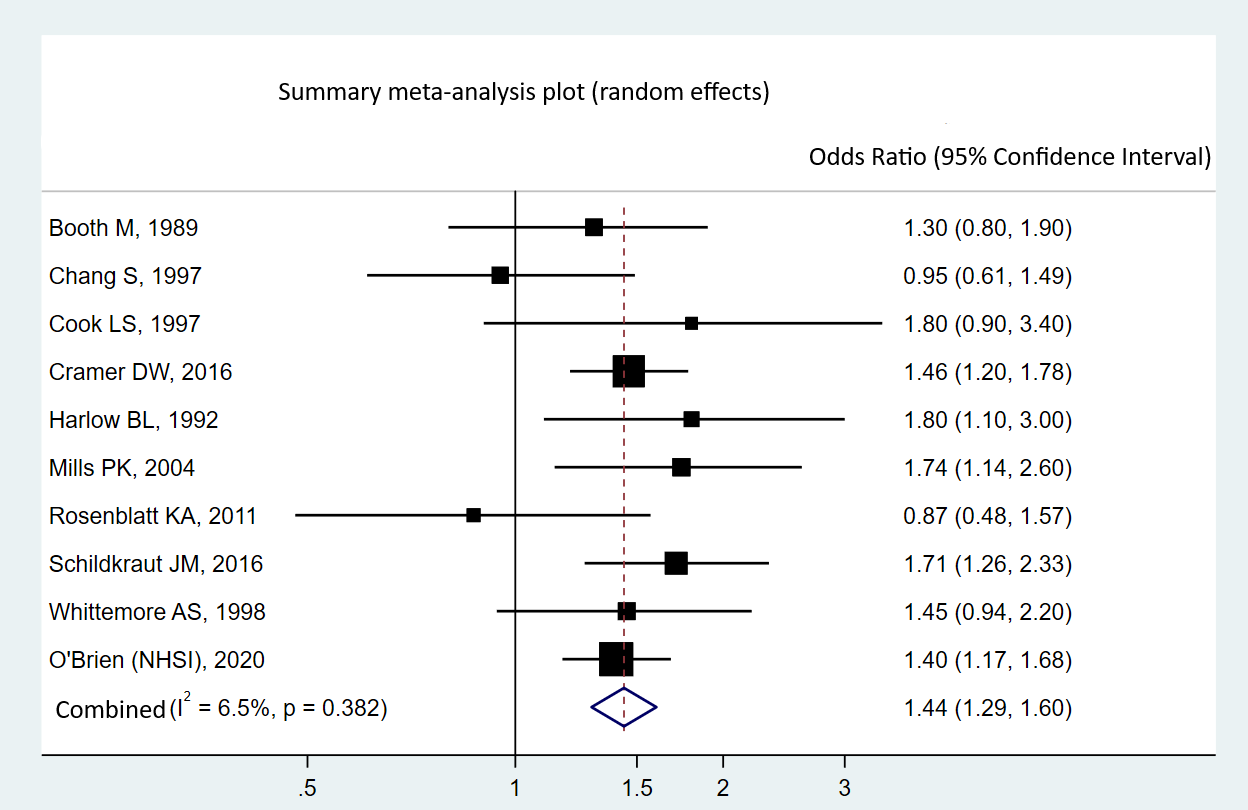
**

**Supplementary Figure 4.** Funnel plot for the risk of publication bias after removing Wu et al.


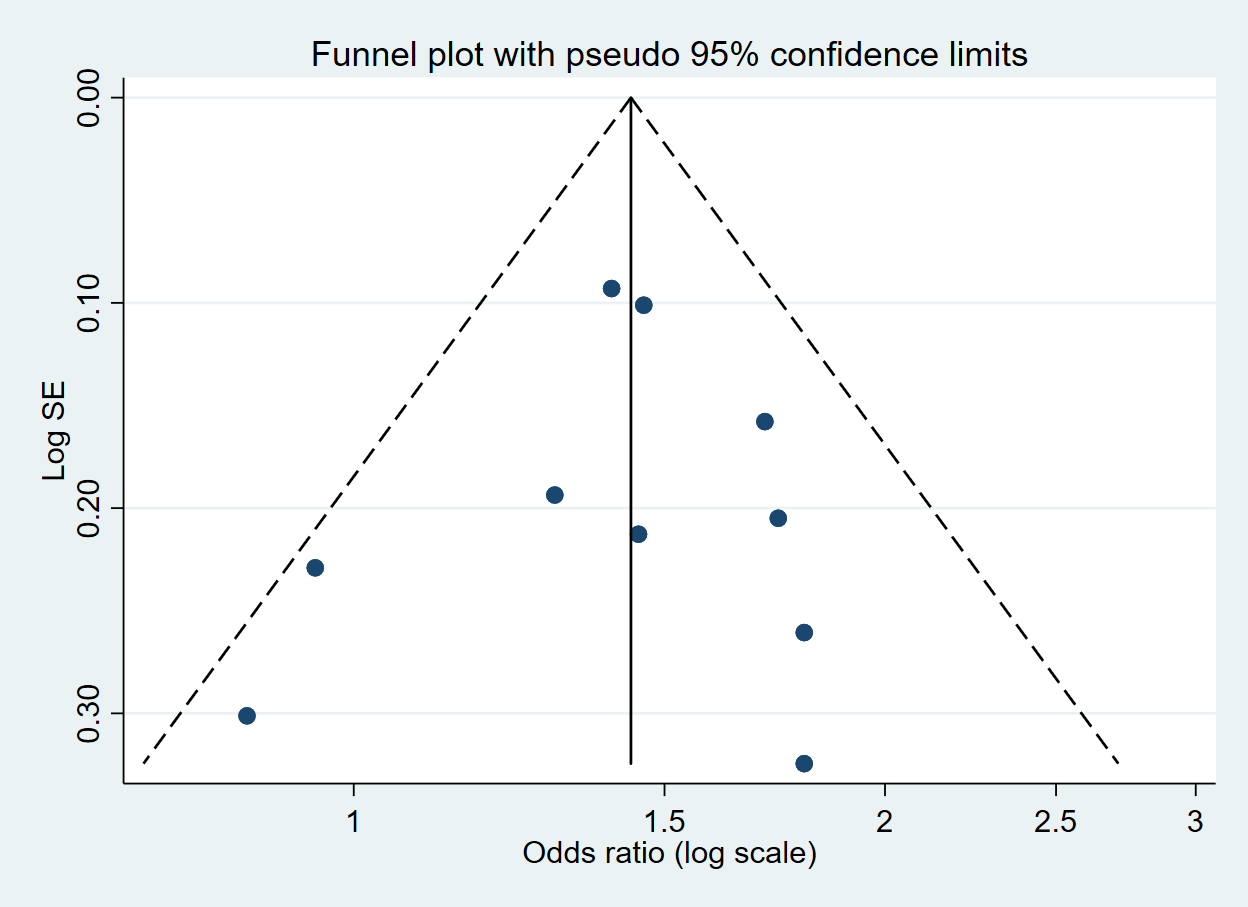


**Supplementary Figure 5**. Forest Plot Showing the Summary Meta-analytic Estimate for the Association Between Frequent Use of Perineal Talcum Powder Products and The Risk of Ovarian Cancer after removing the study with the lowest quality score (Booth et al.) for a sensitivity assessment. The pooled odds ratios and 95% confidence interval are on the right side of the plot.

**
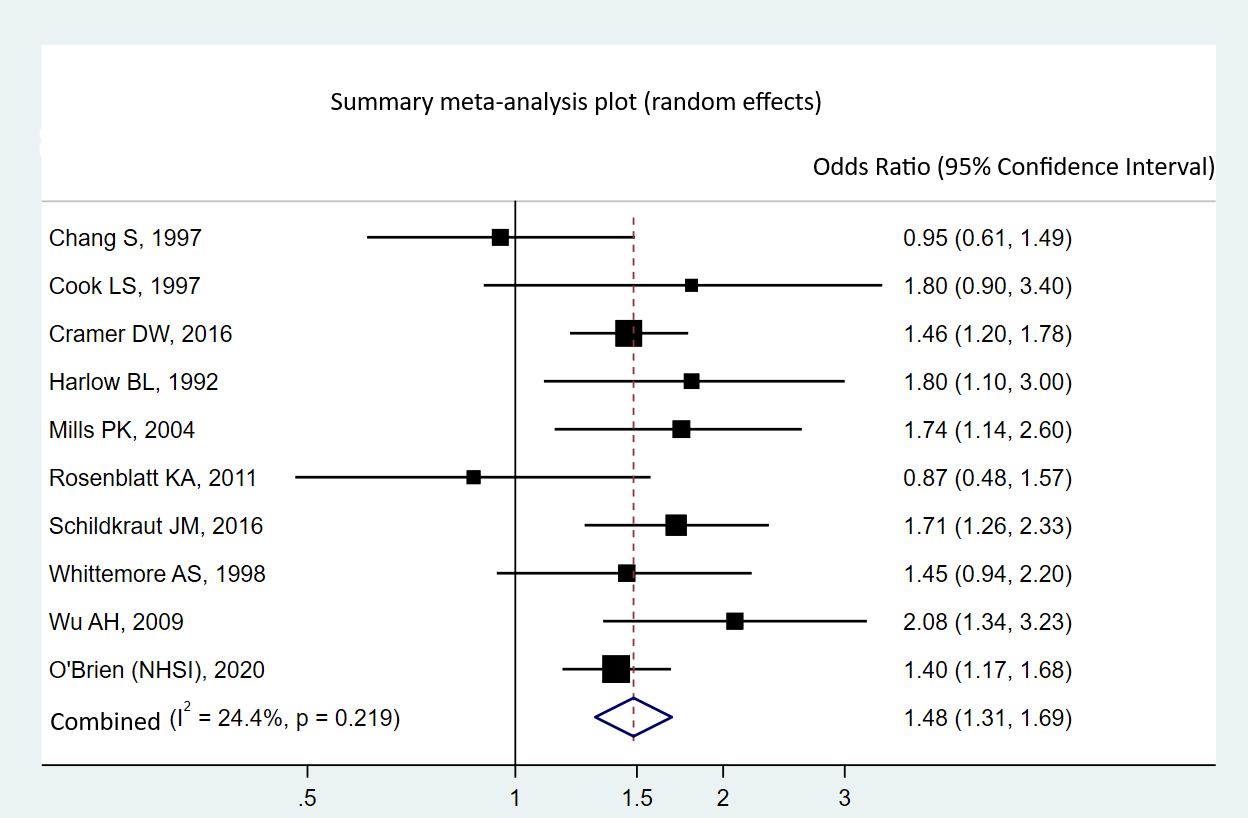
**

**Supplementary Figure 6**. Funnel plot for the risk of publication bias after removing Booth et al.

**
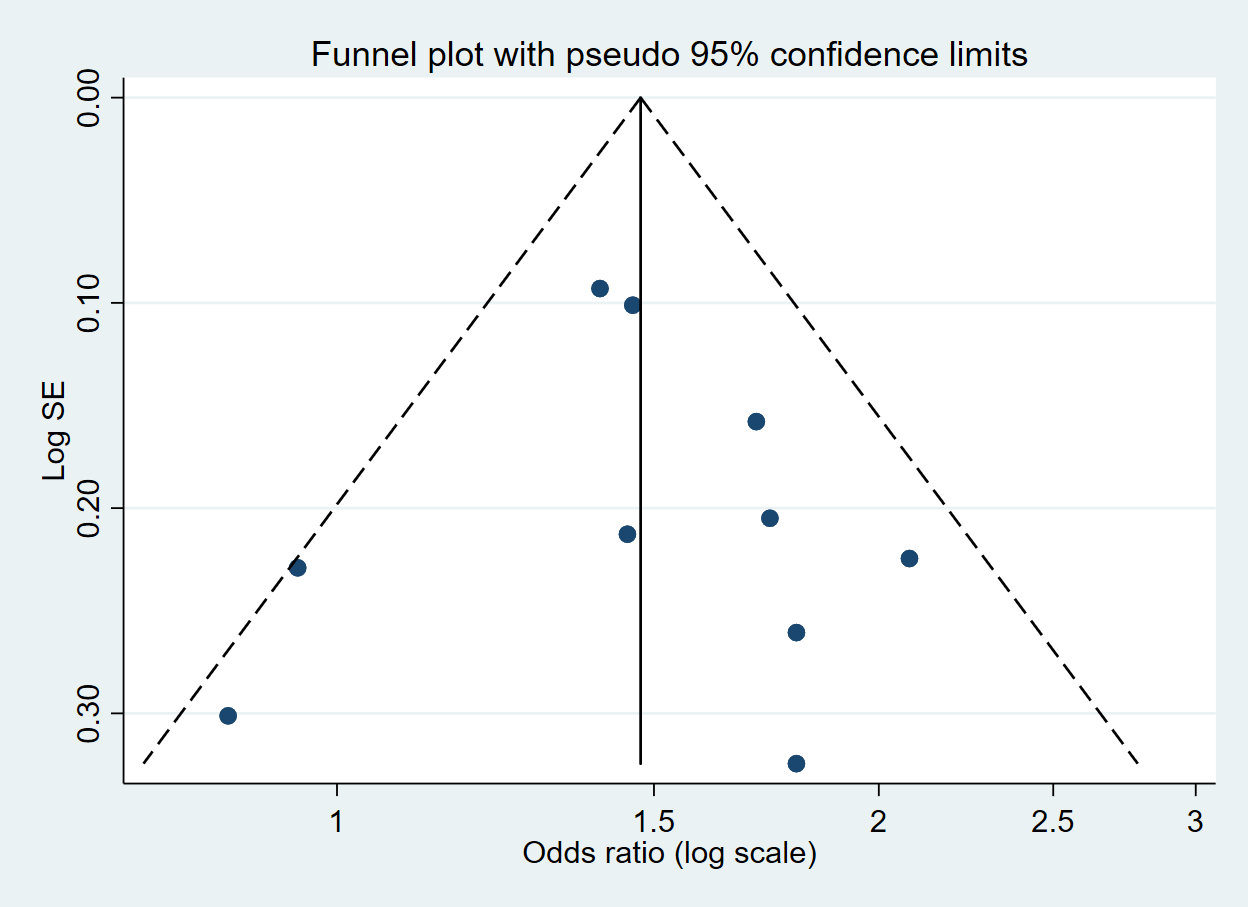
**

**Supplementary Figure 7**. Forest Plot Showing the Summary Meta-analytic Estimate for the Association Between Frequent Use of Perineal Talcum Powder Products and The Risk of Ovarian Cancer after removing the three studies with the lowest quality scores, Booth et al, Cook et al, and Whittenmore et al. The pooled odds ratios and 95% confidence interval are on the right side of the plot.


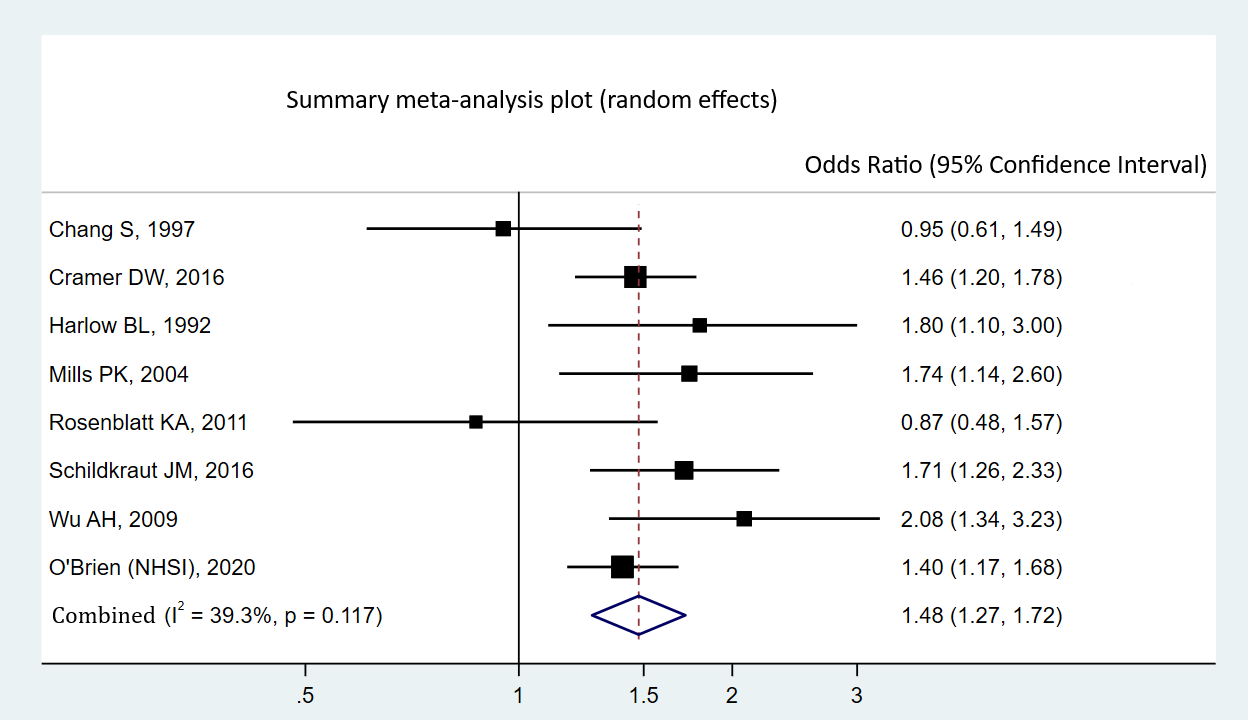


**Supplementary Figure 8.** Funnel plot for the risk of publication bias after removing Booth et al, Cook et al, and Whittenmore et al.


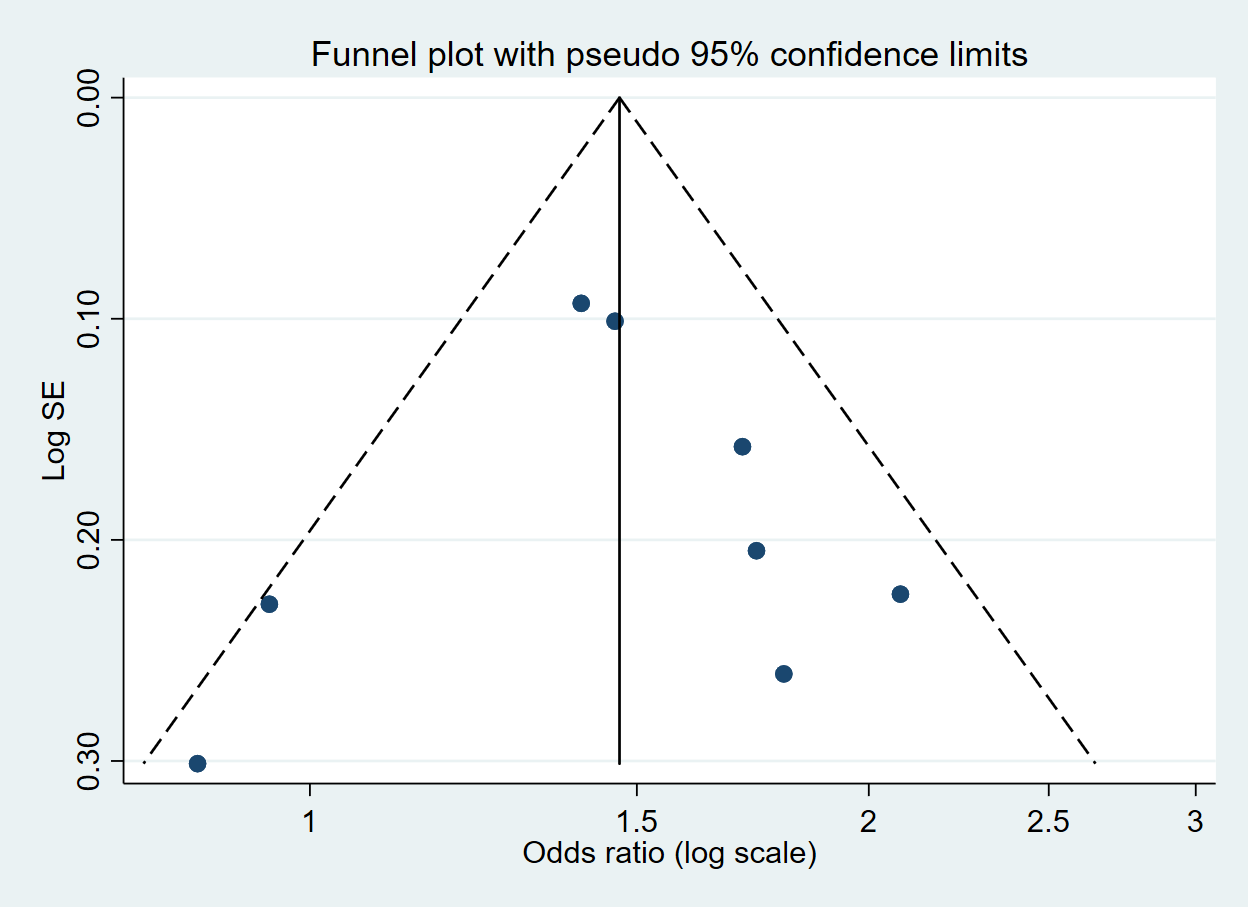

Supplement: Supplementary file 1 — (DOCX 13883 kb) [file 11606_2022_7414_MOESM1_ESM.docx]
